# Migration of Crab Plovers *Dromas ardeola* Wintering at Barr Al Hikman, Oman

**DOI:** 10.1002/ece3.71917

**Published:** 2025-08-05

**Authors:** Roeland A. Bom, Andy Y. Kwarteng, Jan A. van Gils

**Affiliations:** ^1^ Department of Coastal Systems NIOZ Royal Netherlands Institute for Sea Research AB Den Burg Texel the Netherlands; ^2^ BirdEyes, Centre for Global Ecological Change at the Faculties of Science & Engineering and Campus Fryslân, University of Groningen Leeuwarden the Netherlands; ^3^ Department of Geological Engineering University of Mines and Technology Tarkwa Ghana; ^4^ Groningen Institute for Evolutionary Life Sciences (GELIFES) University of Groningen Groningen the Netherlands

**Keywords:** Arabian Peninsula, breeding colonies, GPS tracking, stopovers

## Abstract

Crab plovers *Dromas ardeola* are shorebirds endemic to the coasts of the Indo‐West Pacific biogeographical area. Very little is known about the migration of this enigmatic bird. Here, we studied the migratory itineraries of six crab plovers tracked between their wintering grounds in Barr Al Hikman, Oman, and their breeding grounds on islands in the north‐west of the Arabian/Persian Gulf in Iran and Kuwait. During spring migration, all tagged birds followed a similar route. On the first leg, birds flew north over desert and mountain areas. After reaching the Arabian/Persian Gulf, they followed the northern coastline, where most birds had several stops. Birds initiated spring migratory flight between February 28 and May 7, and it took 3–24 days to travel from the non‐breeding to the breeding areas. The birds spent between 96 and 174 days at the breeding colonies. Autumn migratory flights were mostly along the eastern coast of the Arabian/Persian Gulf. Four birds followed a coastal route back to Barr Al Hikman, similar to spring migration. Two birds followed a southerly route and short‐cut the last stretch of 500 km by crossing the Empty Quarter (Rub Al Khali) desert region. Autumn migratory flights occurred between July 19 and October 24, lasting 3–91 days. Most migratory flights occurred predominantly between 6 p.m. and 6 a.m. and were almost always less than 25 m altitude above the Earth's surface. The maximum height of 1748 m above sea level was measured above the Oman Mountains. Crab plovers were able to cross potential barriers (i.e., the two desert areas and a mountain range) within a single night. We discuss our results with respect to the migratory connectivity known for this species.

## Graphical abstract

Crab plovers *Dromas ardeola* are unique shorebirds endemic to the Indo‐West Pacific, yet their migratory patterns remain poorly understood. This study provides the first detailed insights into the migratory itineraries of crab plovers between their wintering grounds in Oman and breeding sites in Iran and Kuwait. Our findings reveal consistent spring and autumn migration routes, including crossings over deserts and mountains, as well as a large number of stopping sites along the way.

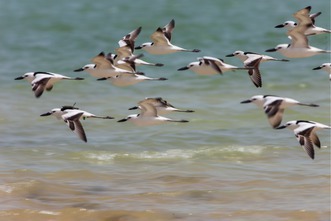

## 1 Introduction

**FIGURE 1:**
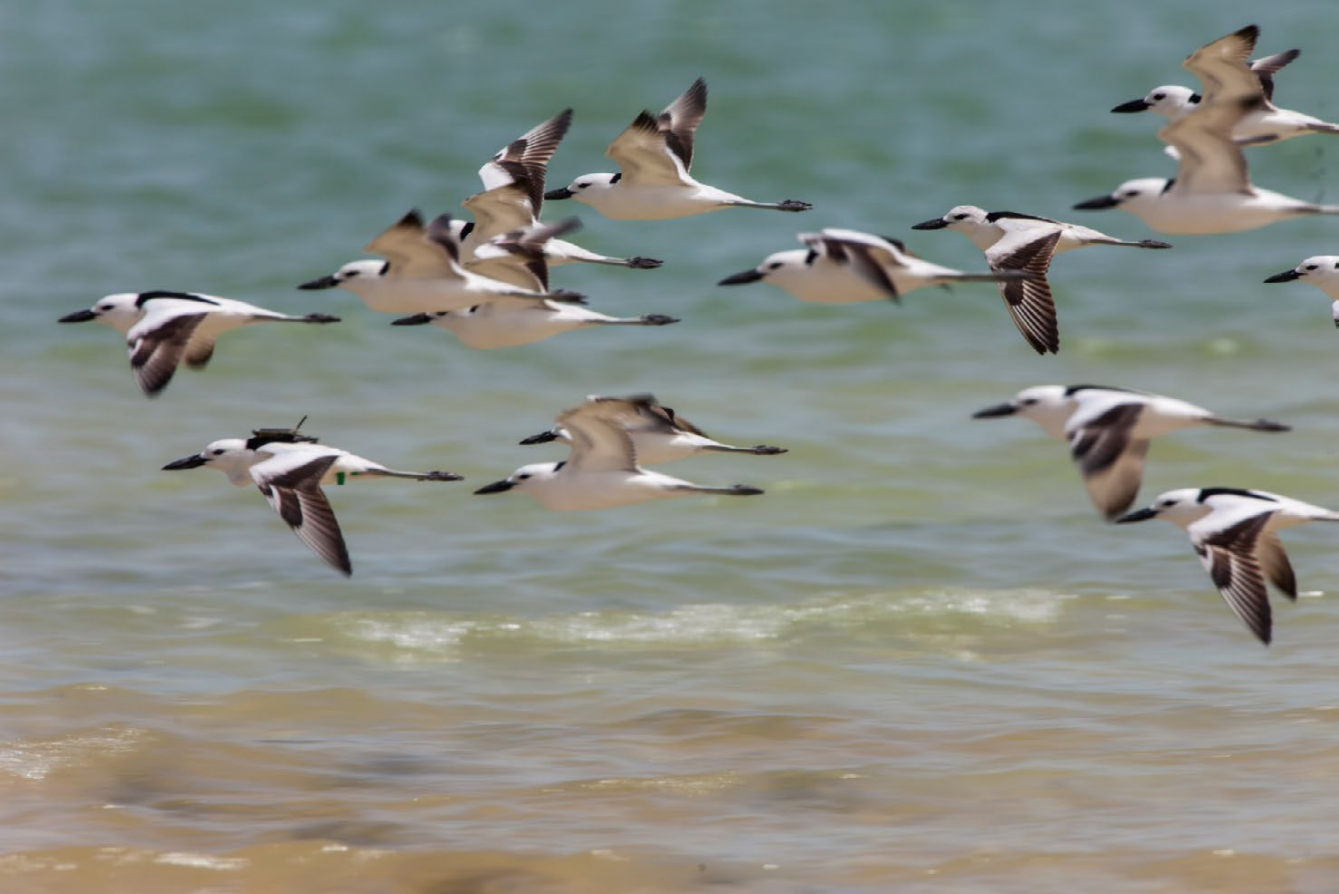
A group of crab plovers at Barr Al Hikman. The second bird from the left carries a tag.

The crab plover *Dromas ardeola* is a monotypic wader found on desert coasts (Figure 1). Its nesting areas are confined to a few colonies, estimated to be 56, in the north‐western Indian Ocean and the Red Sea (Aspinall and Hockey 1996; Bom and Al‐Nasrallah 2015). Its wintering range extends to most of the shores of the Indo‐West Pacific biogeographical area. The species is relatively understudied, with most research focusing on its intriguing nesting behavior. Crab plovers are the only known waders that breed in self‐excavated burrows, approximately two meters long, where they lay a single egg. They attend their egg very little, presumably because the temperature inside the burrows is near‐optimal for incubation (Aspinall and Hockey 1996; De Marchi et al. 2008, 2015).

The migratory movements of crab plovers remain largely unknown. The published study of a tracked crab plover shows how a bird migrated between breeding areas in the United Arab Emirates (UAE) to wintering areas on Aldabra, Seychelles (Javed et al. 2011). Furthermore, resightings of colour‐ringed birds show the connectivity between the Sultanate of Oman and breeding colonies in the Arabian/Persian Gulf (Bom 2019), and a ringed bird in Tanzania suggests connectivity with the breeding colonies in the Red Sea (Hagemeijer et al. pers comm). Likewise, there are a few observations on the species' migration flight. Conceivably, the species may follow a coastal route and avoid large desert areas, as crossing these areas may impose heat stress, especially during the day. Nevertheless, intriguing reports exist of crab plovers flying over the desert in the middle of the night (Aspinall and Jennings 2010). Possibly, crab plovers may be able to cross these areas during the night to prevent them from overheating.

Currently, the IUCN status of crab plovers is “Least Concern” (BirdLife International 2024). However, conservation concerns are often raised given that the species moves in a rapidly changing world marked by habitat loss, pollution, overfishing, and climate warming (Sale et al. 2011; Chapman 2017; Bom et al. 2018). Establishing the connectivity between breeding and non‐breeding areas may help understand current and future population dynamics in the species. Likewise, a description of migration ecology and habitat use may provide important basic information on the ecology of this enigmatic species.

Here, we study the connectivity and migration ecology of crab plovers, on the basis of six individuals that were tagged at their wintering grounds in Barr Al Hikman, Sultanate of Oman. This RAMSAR area hosts up to 8759 individuals representing 10%–15% of the estimated population and is the most important wintering ground for the species (Delany et al. 2009; de Fouw et al. 2017). We establish the connectivity between Barr Al Hikman and breeding areas, provide a detailed picture of the migration routes, and highlight the crossings of potential barriers. Finally, we describe the phenological timing, stopover site use, and migratory flight times and altitudes.

## 2 Methods

### 2.1 Study Area

**FIGURE 2:**
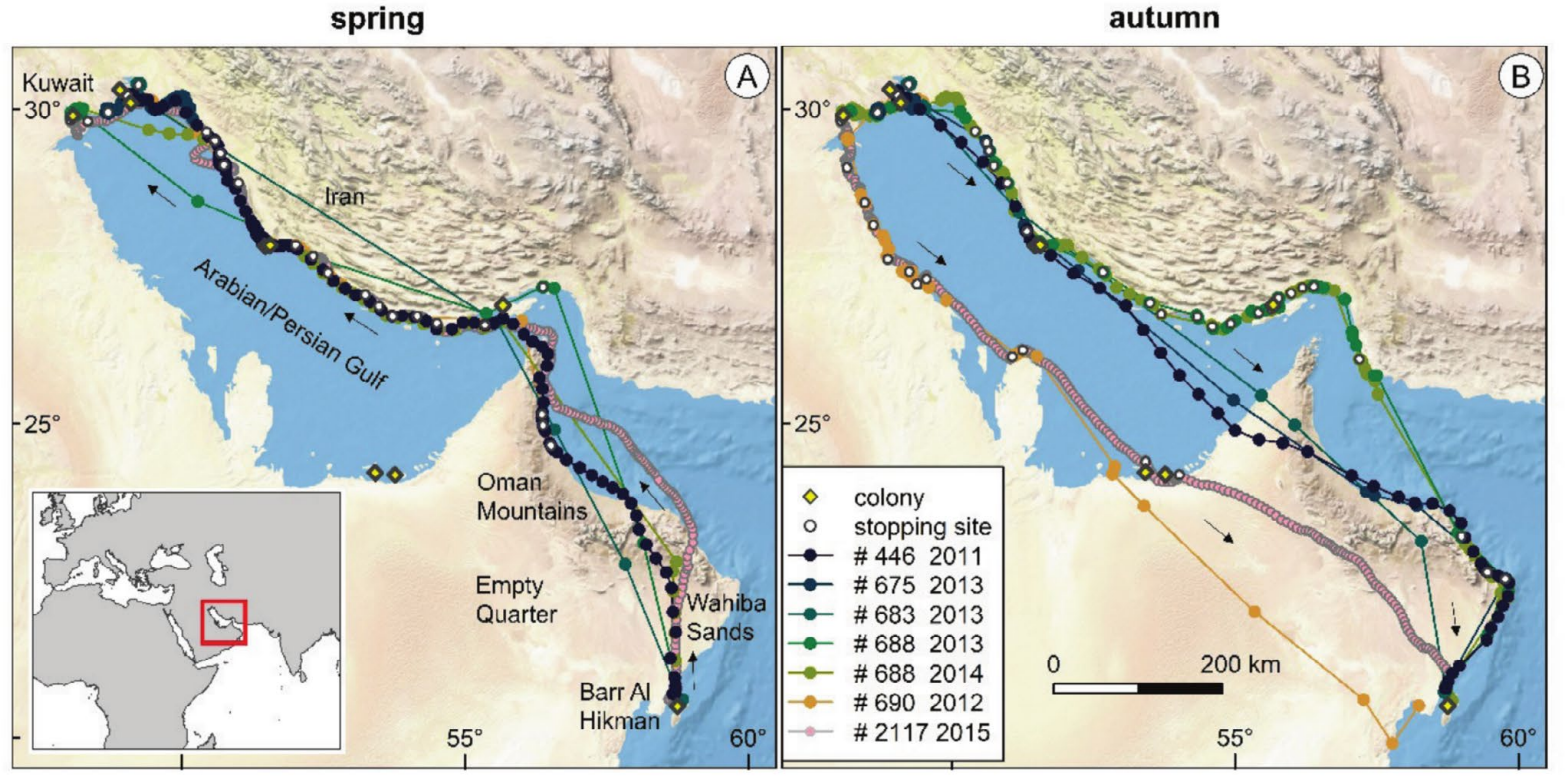
(A) Spring and (B) Autumn migration of the six crab plovers tagged at Barr Al Hikman, Sultanate of Oman in 2011–2014′, including the 2‐year tracks of #688. The small arrows highlight the travel direction. The legend refers to the tag label and year of tracking. Stopping sites and breeding colonies (from Bom and Al‐Nasrallah 2015) are also shown as well as places and areas mentioned in the manuscript. Note the crossing of the Oman Mountains and Wahiba Sands in spring and the crossings of the Empty Quarter in autumn.

Barr Al Hikman peninsula is approximately 900 km^2^ in area and lies in the central‐eastern part of the Sultanate of Oman, 25 km west of Masirah Island (Figure 2). During low tides, approximately 190 km^2^ of intertidal mudflats are exposed on the east and southwest coast of the peninsula. The wetland is among the world's most undisturbed tropical intertidal ecosystems (de Fouw et al. 2017).

### 2.2 Tracking Details

Crab plovers were caught with mist nets at night. In April 2011, November 2012, and November 2014, 3 (all males), 8 (4 males, 4 females), and 10 (7 males, 3 females) adult crab plovers respectively, were fitted with UvA BiTS trackers (Figure 1; Bouten et al. 2013), using a full‐body harness made of 6 mm wide Teflon strings and weighing about 2 g. In addition, all birds received a unique combination of colour rings (Bom et al. 2018). Birds were released within 40 min after capture. The tracked crab plovers weighed an average of 340 g (SD ±8 g) in 2011, 380 g (SD ±38 g) in 2012, and 367 g (SD ±41 g) in 2014. The mean weight of the trackers and their attachments was 15.0 g (SD ±0.5 g) in 2011, 15.1 g (SD ±0.5 g) in 2012, and 9.8 g (SD ±0.1 g) in 2014. This means that, on average, the birds had to cope with 3%–4% added mass. Additional information on the tracked crab plovers is presented in Table S1.

The tracking device was solar‐powered and included a GPS receiver (for geographic position, instantaneous speed and altitude) and a tri‐axial accelerometer. When tags were within reach of the antenna network, both the logging and transmission intervals could be changed. Generally, the trackers were set to measure positions every 10 min. The tags were programmed to switch to lower logging intervals when full memory was reached. On the basis of the locations from a breeding pair of European Honey Buzzard *Pernis apivorus* with a known nest location, the tags were measured to have a positional mean error of 67 m and a mean altitude error of 21 m (Bouten et al. 2013).

We retrieved year‐round tracking data for six tags. Among these, one tagged individual (#688) was tracked for two consecutive years. The tracking interval differed among individuals because of differences in free memory capacity that ranged from a fixed interval of 10 min (tag #2117) to 6 h (all other tags).

Four other individuals with trackers were observed for at least a year after tag deployment, but no data could be downloaded, and that was most likely because of tag failure. The other 11 birds were observed and tracked the first month after tagging, but were not observed or tracked the following year when we returned to the field.

## 3 Data Analysis

We first describe the general migration routes of the six tracked crab plovers with a focus on crossing potential geographical barriers, including: (i) the Oman Mountains (Hajar Mountains), a 700 km wide and up to 3009 m high mountain range ~300 km north of Barr Al Hikman; (ii) the Wahiba Sands (Sharqiya Sands), a relatively small desert area of 12,500 km^2^, directly north of Barr Al Hikman; and (iii) the Empty Quarter (Rub Al Khali), a large desert area of 650,000 km^2^, covering a large part of the Arabian Peninsula and bordering the western part of Barr Al Hikman. The average daytime temperatures at the weather station in Shaybah, Saudi Arabia, in the Empty Quarter are 25°C and 35°C in spring and autumn, respectively (https://www.timeanddate.com/weather/@392477/climate, accessed on 2025‐05‐30).

Next, we examined the minimum distance traveled between winter and breeding areas. We calculated the total distance traveled by each individual by summing the distances between consecutive GPS points using the Geodist function (Padgham and Sumner 2021) in the R software (R Development Core Team 2023). We further identified stopping sites during migration by employing a hierarchical density‐based clustering approach using the ‘dbscan’ function from the HDBSCAN package in R (Hahsler et al. 2019). HDBSCAN detects clusters of spatial points on the basis of two parameters: the minimum number of points required to form a cluster (MinPts) and the radius of the neighborhood (*ε*) (Kwon and Kempenaers 2023). For this analysis, we considered only stationary points—those with an instantaneous speed of less than 3.4 m s^−1^ (Shamoun‐Baranes et al. 2012), with all other points categorized as flight.

We determined the phenological timing of departure and arrival at both wintering and breeding sites. The first point outside Barr Al Hikman or the breeding areas was defined as the departure, whereas the first point inside these areas marked the arrival. For the individual tracked across two consecutive years, we assessed between‐year consistency in migration routes and timing.

We further examined the day‐night timing of migratory flight. All flight points between departure and arrival at breeding and wintering sites and more than 5 km away from any stopping site were considered as migratory flight positions. Day and night periods were distinguished using the ‘sunRiseSet’ function from the ‘suncalc’ package in R (Thieurmel and Elmarhraoui 2022).

We finally investigated flight altitude relative to ground level. This was done by subtracting the Earth's elevation from each recorded flight position. Elevation data were retrieved using the ‘get_elev_point’ function from the ‘elevatr’ package in R (Hollister et al. 2017). Throughout the manuscript, we refer to migratory events prior to and after breeding as spring and autumn migration, respectively.

## 4 Results

### 4.1 Migration Route

During spring migration, all birds followed a more‐or‐less similar route (Figure 2). After take‐off from Barr Al Hikman, the crab plovers flew north over the Wahiba Sands and the Northern Oman Mountains to the Straits of Hormuz and to the south coast of the Islamic Republic of Iran. From then onward, they followed a northwest coastal route, making frequent stopovers along the Iranian coast. All birds went to known breeding sites in the northwest corner of the Arabian/Persian Gulf. Four birds spent the breeding season in southwest Iran at Dara Island or Ghabr‐e‐Nakhoda, and two birds on Bubiyan Island in the State of Kuwait. These breeding sites are approximately 1600 km from Barr Al Hikman (the shortest distance). The average spring migration distance was 1788 km (SD = 206 km). The longest distance of 2087 km was recorded by #2117, which also had the highest fix rate, and the shortest distance of 1518 km by #683.

With autumn migration, four birds took a more or less similar route back to Barr Al Hikman, again with frequent stops along the way. Two of these birds crossed the Northern Oman Mountains at similar locations prior to arriving at Barr Al Hikman, whereas the other two circumvented this mountain range. Two birds migrated south along the Arabian/Persian Gulf through Kuwait, Saudi Arabia, Qatar, and the United Arab Emirates and eventually flew approximately 760 and 780 km, respectively, over the Empty Quarter to Barr Al Hikman. The average autumn migration distance was 2046 km (SD = 299). The highest distance of 2455 km was recorded by #2117 and the lowest of 1673 km by #446.

In total, we identified 23 stopping places during spring migration and 37 during autumn migration. Several of these correspond to known breeding areas (Figure 2). The individual #688, with two consecutive years of migration, used essentially the same migration route in both years (Figure 2).

### 4.2 Phenology and Stopping Behaviour

**FIGURE 3:**
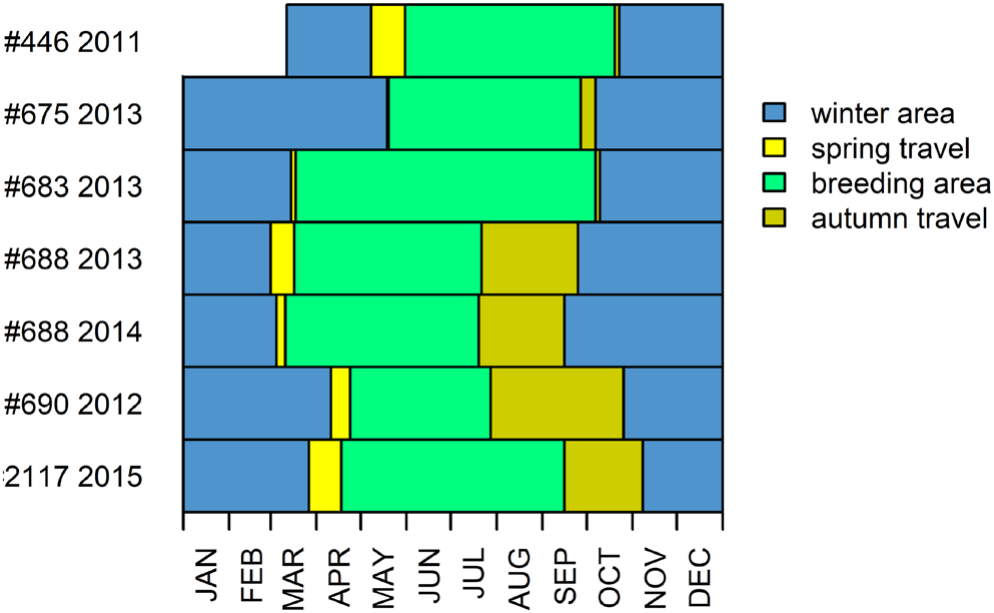
Phenology of six crab plovers tagged at Barr Al Hikman, Sultanate of Oman in 2011–2014. Spring travel was defined as the period between departure from Barr Al Hikman and arrival at the breeding areas. Autumn travel was defined in a similar way. Tag #446 was deployed on 11 March, the other tags in the year prior to the plotted data. The tracked birds showcase a large variation in travel time between winter and breeding areas and vice versa as well as a difference in phenological timing.

In spring, the tagged crab plovers left Barr Al Hikman between February 28 and May 7 (Figure 3), and the travel to the breeding areas lasted between 3 and 24 days (mean = 15 days). The birds arrived at the breeding sites between March 10 and May 30, where they spent between 96 and 174 days (mean = 136 days). The tagged birds left the breeding area between July 19 and October 19. It took most birds longer to travel from the breeding areas to Barr Al Hikman (range 3–91 days, mean = 41 days) than vice versa or the spring migration. The birds arrived back at Barr Al Hikman between September 15 and November 7. The bird that was tracked for two years had similar timing between the years (Figure 3).

Most of the birds made several stops along the routes, both in spring and autumn. In total, we identified 99 stopping events. The bird with tag #683 probably flew straight from the wintering areas to the breeding areas in 3 days. However, because the measuring interval for this tag was 6 h it is associated with some uncertainty. Stopover duration ranged from 10 min to 76 days. The majority (70%) of the stops were less than a day.

### 4.3 Daily Timing of Migratory Flight

**FIGURE 4:**
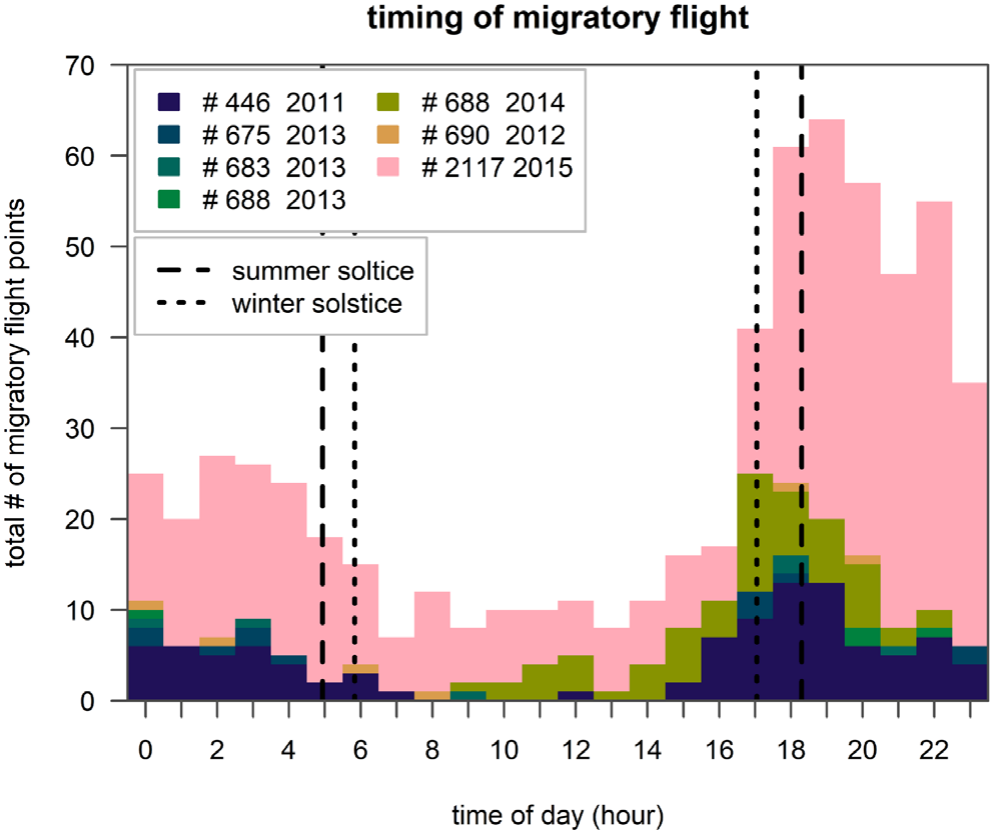
Timing of the migratory flight of six crab plovers tagged at Barr Al Hikman, Sultanate of Oman in 2011–2014. Vertical lines depict sunrise and sunset of summer and winter solstice. Most of the migratory flights are nocturnal.

The migratory flight mostly occurred during the night (Figure 4). Out of the 625 recorded migratory flight points, 82% were sampled between 17:00 and 06:00 and 58% between 17:00 and 24:00. In spring, the crossing over the Wahiba Sands and the Northern Oman Mountains could be measured in detail in three birds; it occurred between 18:50–23:54 (#446, 30 min tracking interval), 19:56–22:31 (#6882013, 30‐min interval) and 17:48–22:27 (#2117, 10‐min interval). The single bird (#683) making the reversed crossing in autumn did this around midnight. The relatively long autumn crossing over the Empty Quarter by the two birds occurred approximately between 18:00 and 6:00 (#690, 6‐h tracking interval) and 18:21 and 06:46 (#2117, 10 min interval).

### 4.4 Relative Flight Altitude

**FIGURE 5:**
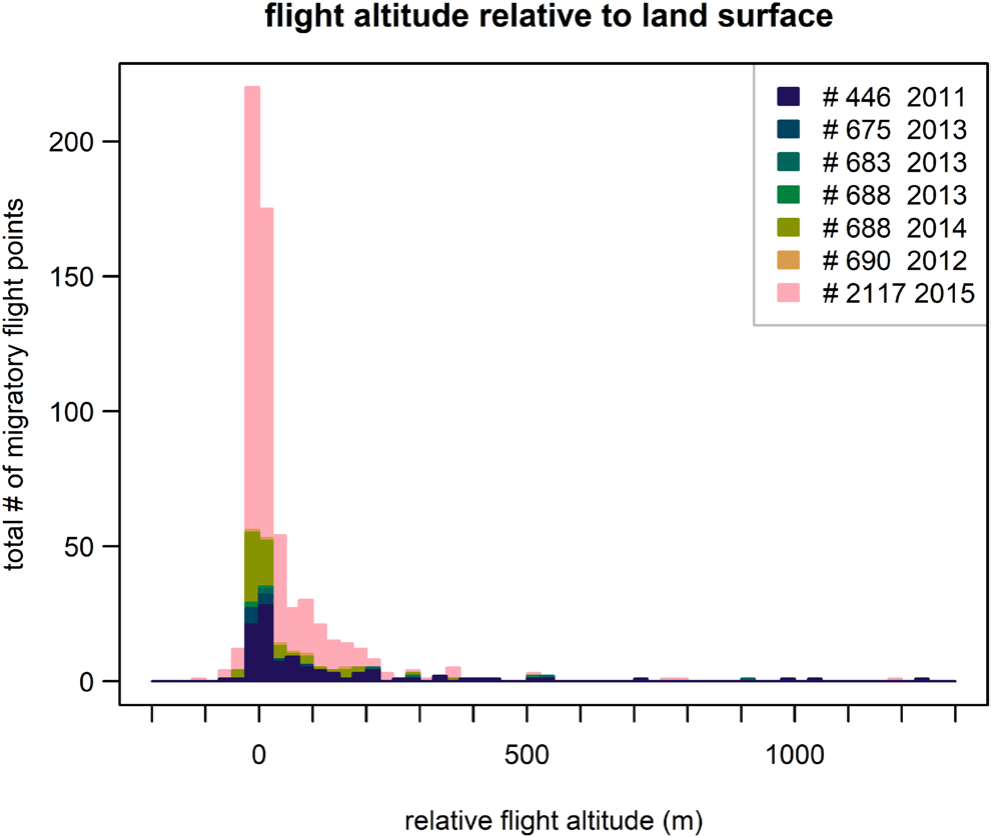
Flight altitude relative to the land surface during the migratory flight in four tracked crab plovers tagged at Barr Al Hikman, Sultanate of Oman in 2011–2014. Most flight altitudes were close to the land surface. The tags were measured to have a mean altitude error of 21 m, which can explain the negative measurements.

**FIGURE 6:**
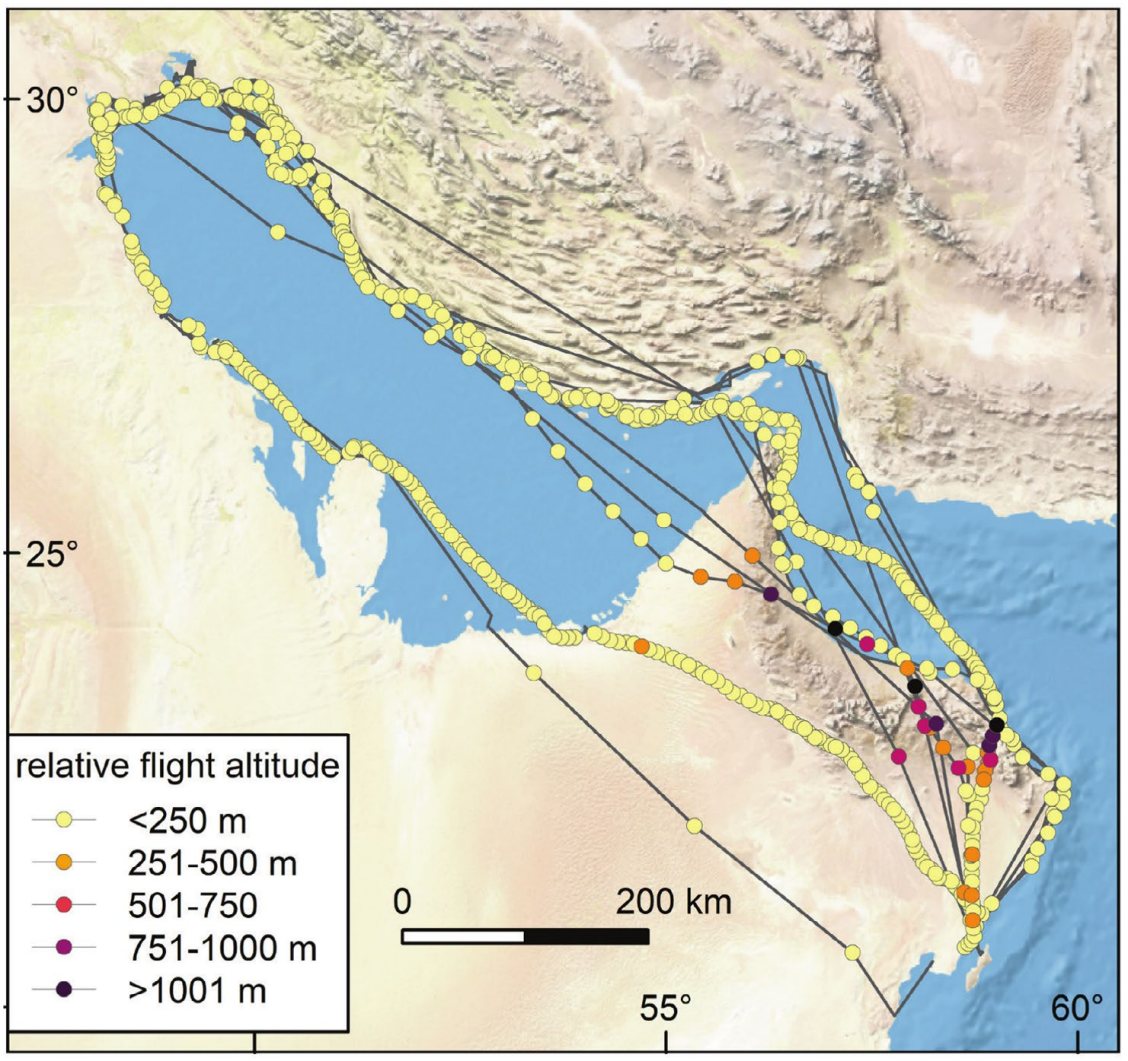
Flight altitude relative to Earth surface for all migratory flight positions. Relatively high flight positions are concentrated around the Oman Mountains.

During the migratory flight, birds flew within 25 m of the Earth's surface during most of their flights (Figure 5). Few positions were recorded with a relative flight altitude above 500 m. High absolute and relative flight altitudes were recorded close to the Oman Mountains (Figure 6). The maximum recorded flight altitude was 1748 m, which was 846 m relative to the land surface. The maximum altitude relative to the land surface was 1238 m, measured in autumn above the Gulf of Oman, just north of the Oman Mountains.

## 5 Discussion

We studied the migration of crab plovers using tracking data from six birds. Before discussing the results on migration, we first address the perhaps relatively low return rate of the tracked birds. In total, 10 birds were recorded the year after tagging, i.e., in addition to the six tagged birds, we observed four other tagged birds 1 year after tagging (probably with malfunctioning tags). On the basis of an annual apparent survival rate of 90% (Bom et al. 2018), we expected more than 10 of the 21 tracked birds to return to the wintering areas in the following year. One possible explanation for this unexpectedly low return rate might be the use of a full‐body harness. After this tracking study was performed (in the years 2011 and 2013), it became clear that in general birds will have a higher return rate when trackers are mounted with a leg loop instead of a full‐body harness (Gould et al. 2024). The low return rate may raise questions about how representative our results are for the migration of crab plovers. Although this question is relevant for many tracking studies, we believe that the obtained data give valuable insight into the migratory behaviour of this species, as discussed below. We also note that the bird with 2 years of tracking was very similar in its timing and route, suggesting normal behaviour for this individual.

Previously, three resightings of ringed crab plovers connected the wintering areas in Barr Al Hikman and breeding in the northwest Arabian/Persian Gulf area (Bom 2019). Our study on crab plovers wintering in Barr Al Hikman emphasizes this connection as all six tracked birds went to these same breeding areas. Three colonies have been found in this upper part of the Arabian/Persian Gulf on Bubiyan Island (State of Kuwait), Dara Island (Islamic Republic of Iran) and Gabr‐e‐Nakhoda (Islamic Republic of Iran) (Tayefeh et al. 2013; Bom and Al‐Nasrallah 2015), with a total number of burrows of about 13,300. Thus, in theory, the entire wintering population at Barr Al Hikman, with a maximum count of 8759 birds, could have their origin in the northwest Arabian/Persian Gulf area. Conversely, not all breeding birds from the northwest Arabian/Persian Gulf area could have their wintering areas in the Sultanate of Oman.

All six tracked birds took a similar route between wintering and breeding areas and back. The tracked birds mostly followed a coastal route and frequently stopped along the way. In fact, almost throughout the entire coast of the Arabian/Persian Gulf, the birds made frequent stops at known areas with mudflats suitable for foraging (Behrouzi‐Rad and Behrouzi‐Rad 2010). Undoubtedly, these mudflats are important for the species to complete its annual cycle and for conservation.

Ecological barriers such as mountain ranges, vast stretches of oceans, and deserts can shape the migration of animals (Alerstam et al. 2003; Newton 2008). For instance, deserts are inhospitable for many species and can cause heat stress in migratory birds, particularly when crossings occur during daylight hours (Sjöberg et al. 2021). Similarly, mountain ranges may impose physical barriers that birds may want to circumvent to avoid going to high altitudes (Newton 2008). Between Barr Al Hikman and the breeding sites, we identified three potential barriers: two desert areas (the Wahiba Sands and the Empty Quarter) and the Northern Oman Mountains. The tracked crab plovers crossed these potential barriers multiple times and seemingly without problems. Importantly, in every recorded instance, the birds traversed the ‘barriers’ during a single night, thereby avoiding daytime heat stress. This pattern supports a previous report of a group of crab plovers flying low over the desert in the middle of the night (Aspinall and Jennings 2010).

Interestingly, crab plovers never crossed the Empty Quarter during spring migration, instead following a longer, more northerly route. In this region, prevailing winds blow from the northwest (Li and Sadr 2023). These infamous ‘Shamal winds’ may prevent crab plovers from crossing the Empty Quarter (in a single night), potentially explaining why they avoid this area in spring. Indeed, the tracked birds experienced more headwinds during spring than in autumn (Figure S1). Additionally, during autumn, birds that followed the northerly coastal route were observed to circumvent the Oman Mountain range. We currently lack a clear explanation for this latter pattern.

Many migratory birds fly during the night, possibly to avoid heat stress and reduce predation risk (Newton 2008; Sjöberg et al. 2021). These factors may also explain why birds with prolonged flights fly at high altitudes during the day (Lindström et al. 2021). Thus, predator avoidance and minimizing evaporative cooling may well explain why the migratory flight of all tracked crab plovers almost exclusively occurred during the night and close to the ground.

The tracked crab plovers had similar migration routes, flight time and flight altitude, but the seasonal timing of migration and the number of stopping places along the way differed markedly between tracked individuals. We lack a good explanation for the differences in migration phenology and stopping places. The large difference in the spring phenology of migration could suggest that there is little selection toward arriving and departing from the wintering areas, and arrival date on the breeding grounds (Nilsson et al. 2013). We also note that some of the tracked crab plovers spent most of their time outside the wintering area of Barr Al Hikman, which contrasts with other shorebird species wintering in the area, which may spend as much as 8 months per year in Barr Al Hikman (Bom et al. 2022).

Although we provide many details on the migration ecology of crab plovers, other aspects remain to be further investigated. Future tracking studies at different sites and updated local population estimates should give a better picture of the migratory connectivity of this enigmatic species. Especially relevant for understanding population dynamics in the species is understanding the connectivity between the spatially segregated breeding areas in the Arabian/Persian Gulf and those in the Red Sea.

## Author Contributions

**Roeland A. Bom:** conceptualization (equal), data curation (equal), formal analysis (equal), methodology (equal), project administration (equal), visualization (equal), writing – original draft (equal), writing – review and editing (equal). **Andy Y. Kwarteng:** conceptualization (equal), formal analysis (equal), funding acquisition (equal), writing – review and editing (equal). **Jan A. van Gils:** funding acquisition (equal), supervision (equal), writing – review and editing (equal).

## Ethics Statement

Catching and tagging of crab plovers was carried out under the permission of the Ministry of Environment and Climate Affairs, under permit numbers 5/2011, 31/2011, and 24/2013. Over the years, a large number of employees from the Ministry joined our fieldwork. The study was supported by the Sultan Qaboos University (SQU), both financially and logistically. Several students from the SQU joined us for several weeks in the field. Two students finished their Master's degree projects under the supervision of the first author.

## Conflicts of Interest

The authors declare no conflicts of interest.

## Supporting information


**Data S1:** ece371917‐sup‐0001‐supinfo.docx.

## Data Availability

The data that supports the findings of this paper can be accessed at https://doi.org/10.25850/nioz/7b.b.xj.

## References

[ece371917-bib-0001] Alerstam, T. , A. Hedenström , and S. Åkesson . 2003. “Long‐Distance Migration: Evolution and Determinants.” Oikos 103: 247–260.

[ece371917-bib-0002] Aspinall, S. J. , and P. A. R. Hockey . 1996. “The Indian Ocean's Crab‐Loving Plover.” Arabian Wildlife 3: 32–35.

[ece371917-bib-0003] Aspinall, S. J. , and M. C. Jennings . 2010. “Atlas of the Breeding Birds of Arabia.” Fauna of Arabia 25: 300–303.

[ece371917-bib-0004] Behrouzi‐Rad, B. , and E. Behrouzi‐Rad . 2010. “Status of the Crab Plover *Dromas ardeola* in Persian Gulf and Oman Sea in the Year 2007.” Journal of Environmental Research and Development 5: 191–203.

[ece371917-bib-0005] BirdLife International . 2024. “Species Factsheet: Crab Plover *Dromas ardeola* .” Accesses on 2025‐06‐01.

[ece371917-bib-0006] Bom, R. A. 2019. “The Discovery of Barr Al Hikman as a Wader‐Hub in the Global Flyway Network.” Wader Study 126: 1–3.

[ece371917-bib-0007] Bom, R. A. , and K. Al‐Nasrallah . 2015. “Counts and Breeding Biology of Crab Plovers *Dromas ardeola* on Bubiyan Islands, Kuwait, in 2012–2014.” Wader Study 122: 212–220.

[ece371917-bib-0008] Bom, R. A. , J. R. Conklin , Y. I. Verkuil , et al. 2022. “Central‐West Siberian‐Breeding Bar‐Tailed Godwits *Limosa lapponica* Segregate in Two Morphologically Distinct Flyway Populations.” Ibis 164: 468–485.

[ece371917-bib-0009] Bom, R. A. , J. A. van Gils , K. Oosterbeek , et al. 2018. “Demography of a Stable Population of Crab Plovers Wintering in Oman.” Journal für Ornithologie 159: 517–525.

[ece371917-bib-0010] Bouten, W. , E. W. Baaij , J. Shamoun‐Baranes , and K. C. J. Camphuysen . 2013. “A Flexible GPS Tracking System for Studying Bird Behaviour at Multiple Scales.” Journal für Ornithologie 154: 571–580.

[ece371917-bib-0011] Chapman, P. M. 2017. “Assessing and Managing Stressors in a Changing Marine Environment.” Marine Pollution Bulletin 124: 587–590. 27760713 10.1016/j.marpolbul.2016.10.039

[ece371917-bib-0012] de Fouw, J. , A. Thorpe , R. A. Bom , et al. 2017. “Barr Al Hikman, a Major Shorebird Hotspot Within the Asian–East African Flyway: Results of Three Winter Surveys.” Wader Study 124: 10–25.

[ece371917-bib-0013] De Marchi, G. , G. Chiozzi , G. Dell'Omo , and M. Fasola . 2015. “Low Incubation Investment in the Burrow‐Nesting Crab Plover *Dromas ardeola* Permits Extended Foraging on a Tidal Food Resource.” Ibis 157: 31–43.

[ece371917-bib-0014] De Marchi, G. , G. Chiozzi , and M. Fasola . 2008. “Solar Incubation Cuts Down Parental Care in a Burrow Nesting Tropical Shorebird, the Crab Plover *Dromas ardeola* .” Journal of Avian Biology 39: 484–486.

[ece371917-bib-0015] Delany, S. , D. Scott , T. Dodman , and D. Stroud . 2009. An Atlas of Wader Populations in Africa and Western Eurasia. Wageningen.

[ece371917-bib-0016] Gould, L. A. , A. D. Manning , H. M. McGinness , and B. D. Hansen . 2024. “A Review of Electronic Devices for Tracking Small and Medium Migratory Shorebirds.” Animal Biotelemetry 12: 11.

[ece371917-bib-0017] Hahsler, M. , M. Piekenbrock , and D. Doran . 2019. “Dbscan: Fast Density‐Based Clustering With R.” Journal of Statistical Software 91: 1–30.

[ece371917-bib-0018] Hollister, J. , T. Shah , A. L. Robitaille , M. W. Beck , and M. Johnson . 2017. “Elevatr: Access Elevation Data From Various APIs.” R Packag. version 0.1.3.

[ece371917-bib-0019] Javed, S. , S. Khan , J. Nazeer , S. Ahmed , and A. Hammadi . 2011. “UAE Crab Plover Goes to Aldabra, Seychelles.” Phoenix 27: 4–5.

[ece371917-bib-0020] Kwon, E. , and B. Kempenaers . 2023. “Lack of Breeding Site Fidelity and Mate Fidelity in an Enigmatic Socially Monogamous Shorebird.” Animal Behaviour 204: 1–12.

[ece371917-bib-0021] Li, Y. , and R. Sadr . 2023. “Atmospheric Turbulent Characteristics Under Summer Shamal in Coastal Qatar.” Journal of Geophysical Research: Atmospheres 128: e2022JD037971.

[ece371917-bib-0022] Lindström, Å. , T. Alerstam , A. Andersson , et al. 2021. “Extreme Altitude Changes Between Night and Day During Marathon Flights of Great Snipes.” Current Biology 31: 3433–3439. 34197730 10.1016/j.cub.2021.05.047

[ece371917-bib-0023] Newton, I. 2008. The Migration Ecology of Birds. Academic Press, Elsevier.

[ece371917-bib-0024] Nilsson, C. , R. H. G. Klaassen , and T. Alerstam . 2013. “Differences in Speed and Duration of Bird Migration Between Spring and Autumn.” American Naturalist 181: 837–845. 10.1086/670335 23669545

[ece371917-bib-0025] Padgham, M. , and M. D. Sumner . 2021. “Geodist: Fast, Dependency‐Free Geodesic Distance Calculations.” R Packag. version 0.0.7.

[ece371917-bib-0026] R Development Core Team . 2023. “R: A Language and Environment for Statistical Computing.” R Version 4.3.1.

[ece371917-bib-0027] Sale, P. F. , D. A. Feary , J. A. Burt , et al. 2011. “The Growing Need for Sustainable Ecological Management of Marine Communities of the Persian Gulf.” Ambio 40: 4–17. 21404819 10.1007/s13280-010-0092-6 PMC3357718

[ece371917-bib-0028] Shamoun‐Baranes, J. , R. Bom , E. E. van Loon , B. J. Ens , K. Oosterbeek , and W. Bouten . 2012. “From Sensor Data to Animal Behaviour: An Oystercatcher Example.” PLoS One 7: e37997. 22693586 10.1371/journal.pone.0037997 PMC3365100

[ece371917-bib-0029] Sjöberg, S. , G. Malmiga , A. Nord , et al. 2021. “Extreme Altitudes During Diurnal Flights in a Nocturnal Songbird Migrant.” Science 372: 646–648. 33958477 10.1126/science.abe7291

[ece371917-bib-0030] Tayefeh, F. H. , M. Zakaria , G. De Marchi , et al. 2013. “Breeding Biology of the Crab Plover *Dromas ardeola* on the Mond Islands, Northern Persian Gulf, Iran.” Waterbirds 36: 448–462.

[ece371917-bib-0031] Thieurmel, B. , and A. Elmarhraoui . 2022. “_suncalc: Compute Sun Position, Sunlight Phases, Moon Position and Lunar Phase_.” R package version 0.5.1. https://CRAN.R‐project.org/package=suncalc.

