# Supplementary material for: Migration of Crab Plovers Dromas ardeola Wintering at Barr Al Hikman, Oman
Source: Ecol Evol. 2025 Aug 5;15(8):e71917. doi: 10.1002/ece3.71917 (PMC12325891; doi:10.1002/ece3.71917)
Supplement: Supplementary file 1 — Data S1: ece371917‐sup‐0001‐supinfo.docx. [file ECE3-15-e71917-s001.docx]

**Supplementary Material for:**

**Migration of crab plovers *Dromas ardeola* wintering at Barr Al Hikman, Oman**

Roeland A. Bom^1,2^, Andy Y. Kwarteng^3^, Jan A. van Gils^1,4^

^1^NIOZ Royal Netherlands Institute for Sea Research, Department of Coastal Systems, P.O. Box 59, 1790 AB Den Burg, Texel, The Netherlands

^2^BirdEyes, Centre for Global Ecological Change at the Faculties of Science & Engineering and Campus Fryslân, University of Groningen, Zaailand 110, 8911 BN Leeuwarden, The Netherlands

^3^Department of Geological Engineering, University of Mines and Technology, P.O. Box 237, Tarkwa, Ghana

^4^Groningen Institute for Evolutionary Life Sciences (GELIFES), University of Groningen, PO Box 11103, Groningen 9700 CC, the Netherlands

**Table S1**

Characteristics of tagged crab plovers at Barr Al Hikman

| Catch Date | Tag ID | Latitude ºN | Longitude ºE | Ring no. | Sex | Age | Wing  (mm) | Weight  (g) | Bill length (mm) |
| --- | --- | --- | --- | --- | --- | --- | --- | --- | --- |
| 11-Mar-2011 | 446 | 20.73321 | 58.70399 | 5425718 | M | 2 CY | 225 | 335 | 59.6 |
| 13-Nov-2012 | 675 | 20.70770 | 58.67476 | 5425784 | F | Ad | 205 | 360 | 60.4 |
| 18-Nov-2012 | 683 | 20.70295 | 58.66809 | 5425792 | F | Ad | 205 | 362 | 56 |
| 18-Nov-2012 | 688 | 20.70295 | 58.66809 | 5425793 | M | Ad | 215 | 401 | 58.7 |
| 27-Nov-2011 | 690 | 20.73321 | 58.70399 | 5425773 | M | Ad | * | 431 | 65.6 |
| 16-Nov-2014 | 2117 | 20.69679 | 58.66011 | 5503544 | F | Ad | * | 372 | 59.5 |

| * Bird was moulting and therefore wing could not be measured | | |
| --- | --- | --- |
| 2 CY= 2nd calendar year, Ad = adult |  |  |

***Figure S2***

*We calculated flow assistance by relating the travel direction of the bird at each location fix to windspeed and wind direction at that position at the fix time. Travel direction of the bird at a position fix was calculated as the direction between that fix and the next fix using the geosphere R package* (Hijmans *et al.* 2017) *. We retrieved the u and v-wind components* *from the ERA5 wind database* (Hersbach *et al.* 2020)*. We downloaded the ERA5 data (resolution = 0.25° x 0.25°) for the time and space in question at 1 hrs interval and at 100 m altitude from* [*https://cds.climate.copernicus.eu*](https://cds.climate.copernicus.eu)*. Subsequently, each position fix was matched to the ERA5 dataset via nearest-neighbour search based on location and time using the RANN package in R* (Jefferis & Kemp 2025)*. Using the matched u and v-wind components, the wind speed and direction could be calculated at each position fix. Finally, flow assistance was calculated using the function NCEP.Tailwind from the R-package RNCEP* (Kemp *et al.* 2012)*.*

*
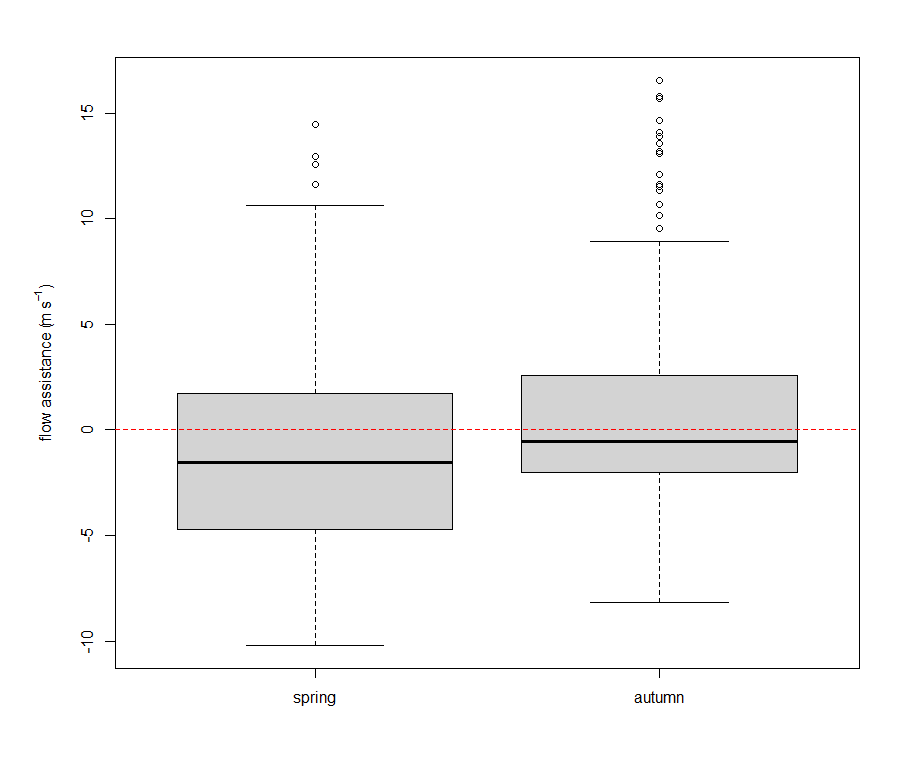
*

*Figure 1. Flow assistance during the spring and autumn migratory flights. Flow assistance values below 0 means head winds and above 0 means tailwind. Birds experience significantly* *less flow assistance in spring than in autumn (linear mixed-effects model with tag as random effect fitted in R using the lmer() function from the lme4 package* (Bates *et al.* 2018)(Bates *et al.* 2018)*. Estimate = 2.29 m s⁻¹, SE = 0.35, t = 6.56, p < 0.0001).*

**References**

**Bates, D., Maechler, M., Bolker, B., Walker, S., Christensen, R.H.B., Singmann, H., Dai, B., Scheipl, F., Grothendieck, G. & Green, P.** 2018. Package ‘lme4’. *Version* **1**: 17.

**Hersbach, H., Bell, B., Berrisford, P., Hirahara, S., Horányi, A., Muñoz‐Sabater, J., Nicolas, J., Peubey, C., Radu, R. & Schepers, D.** 2020. The ERA5 global reanalysis. *Q. J. R. Meteorol. Soc.* **146**: 1999–2049.

**Hijmans, R.J., Williams, E., Vennes, C. & Hijmans, M.R.J.** 2017. Package ‘geosphere’. *Spherical trigonometry* **1**: 1–7.

**Jefferis, G. & Kemp, S.E.** 2025. Package ‘RANN’.

**Kemp, M.U., Loon, E.E. van, Shamoun-Baranes, J. & Bouten, W.** 2012. RNCEP: global weather and climate data at your fingertips.
